# 16S rRNA Gene Amplicon Sequencing of Gut Microbiota from Naked Carp (Gymnocypris przewalskii) in Qinghai Lake, China

**DOI:** 10.1128/MRA.00374-21

**Published:** 2021-06-10

**Authors:** Fayan Wang, Yu Liu, Guangxin Li, Xi Yang, Qiang Gao

**Affiliations:** a State Key Laboratory of Plateau Ecology and Agriculture, Qinghai University, Xining, China; b College of Eco-Environmental Engineering, Qinghai University, Xining, China; Montana State University

## Abstract

Naked carp (Gymnocypris przewalskii) is a second-grade animal under state protection of China. We report 16S rRNA gene amplicon analysis of the gut microbiota of Gymnocypris przewalskii. The three most abundant phyla are *Tenericutes*, *Proteobacteria*, and *Fusobacteria*, and the six most abundant genera are *Aeromonas*, *Clostridium*, *Cetobacterium*, *Shewanella*, *Prochlorococcus*, and *Vibrio*.

## ANNOUNCEMENT

Naked carp (Gymnocypris przewalskii) is an endemic migratory species of Qinghai Lake, which is the largest salt lake in China. The adult fish spawn in freshwater and then return to Qinghai Lake. Research on Gymnocypris przewalskii is of great biological significance for protecting these rare animals and maintaining the ecological balance of Qinghai Lake. The gastrointestinal tract plays critical roles in nutrition, development, immunity, and resistance to invasive pathogens (1). However, our understanding of the intestinal microbiota of this migratory fish is limited. In this study, we analyzed the gut microbiota of Gymnocypris przewalskii during the migratory season. Fish were caught with a trawl net in Qinghai Lake (100.28N, 36.40E) and Buha River (99.78N, 36.72E). Their gut contents were collected and directly conserved in liquid nitrogen until further use. Total DNA was extracted from the gut contents (100 to 200 mg) using the FastDNA spin kit for feces (MP Biomedicals LLC, USA) following homogenization with a homogenizer (Bertin Technologies, France) at 5,000 rpm for 10 min. The V4 region of the gene was amplified using modified primers 515F (5′-GTGYCAGCMGCCGCGGTAA-3′) and 806R (5′-GGACTACNVGGGTWTCTAAT-3′) and TransStart FastPfu DNA polymerase (TransGen Biotech, China) according to the manufacturer’s instructions (2). PCR amplification was performed using the following cycling conditions: 94°C for 5 min, 35 cycles of 94°C for 30 s, 55°C for 30 s, and 72°C for 1 min, and, finally, 72°C for 10 min. The resulting PCR products were examined by 2% agarose gel electrophoresis and further purified using a gel extraction kit (Omega Bio-tek, USA). The libraries were generated using PCR with Illumina adapters connected (New England BioLabs, USA) and were sequenced on the MiSeq PE300 platform (Illumina, San Diego, CA). Library construction and sequencing were performed at Majorbio Bio-Pharm Technology Co. Ltd. (Shanghai, China).

Raw fastq files were demultiplexed and quality filtered with QIIME2 v.2021.2 (3). UPARSE (USEARCH v.11.0) (http://drive5.com/uparse) (4) was utilized to conduct operational taxonomic unit (OTU) clustering analysis at 97% identity. Chimeric sequences were identified and removed using UCHIME v.4.2 (5). Representative sequences of OTUs were picked up against the Silva_132 16S database (http://www.arb-silva.de) to determine taxonomy.

**FIG 1:**
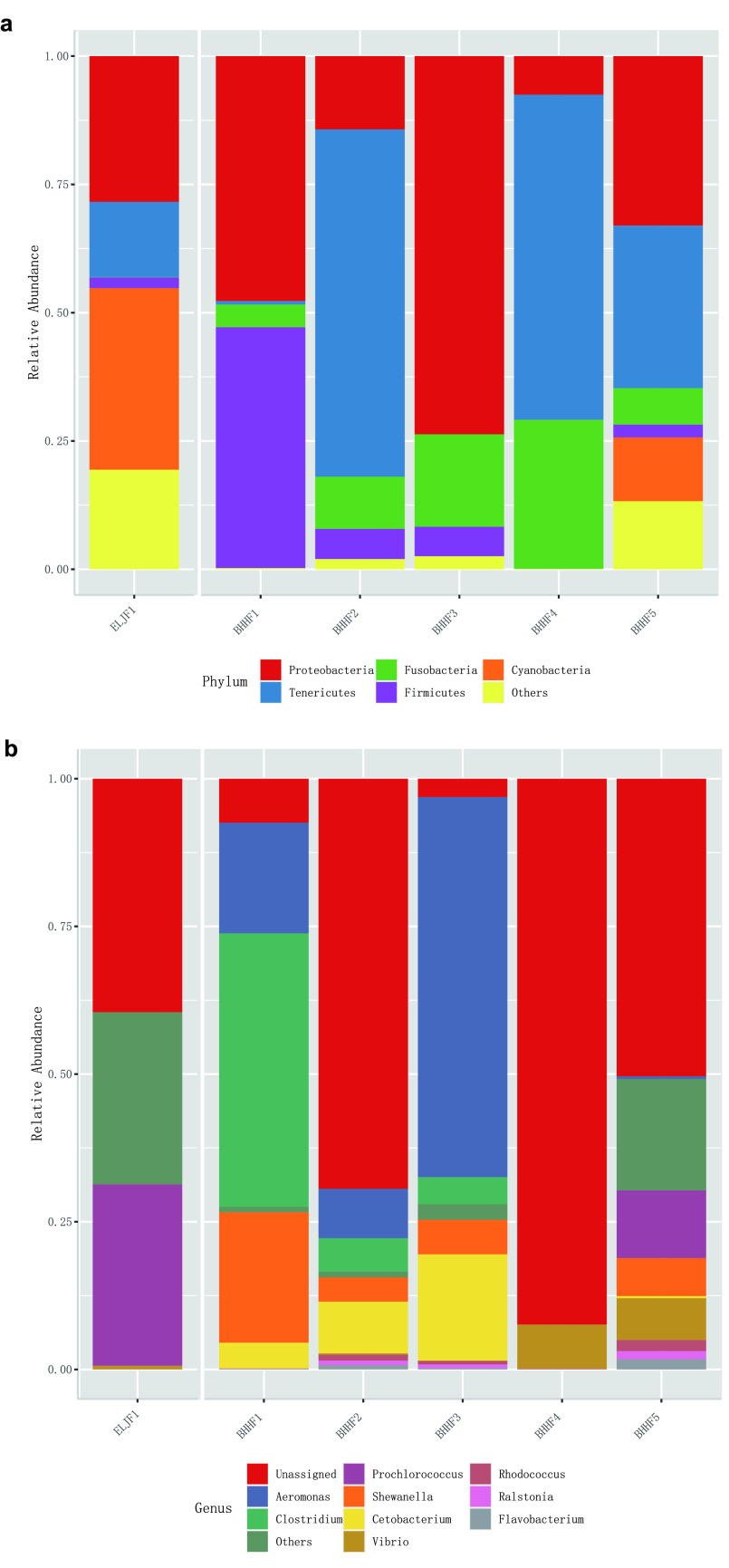
Relative abundance of the top bacterial phyla (a) and genera (b) in samples from different sampling sites.

All statistical analyses were performed with R v.4.0.2 (https://www.r-project.org) and the MicrobiomeAnalyst Web-based tool (https://www.microbiomeanalyst.ca) (6). The total number of reads obtained in this study was 125,158 (Table 1). OTUs were assigned to 16 bacterial phyla, 34 classes, 57 orders, 100 families, and 139 genera. The three most abundant phyla were *Tenericutes* (41.4%), *Proteobacteria* (28.7%), and *Fusobacteria* (15.7%) (Fig. 1a), and the six most abundant genera were *Aeromonas* (15.8%), *Clostridium* (7.8%), *Cetobacterium* (6.1%), *Shewanella* (5.3%), *Vibrio* (3.1%), and *Prochlorococcus* (2.8%) (Fig. 1b). *Aeromonas* strains cause a wide variety of aquaculture animal diseases, not only bringing serious economic losses to the aquaculture industry but also infecting people and animals through aquatic animals and aquatic products, leading to diarrhea and food poisoning (7, 8). The dominance of *Aeromonas* in the gut microbiota suggested that Gymnocypris przewalskii fish were plagued by such pathogens. The findings for other genera and species were similar to the findings of previous studies on the gut microbiota of fishes (9).

**TABLE 1:** Summary of sample data

Sample	Sample site	Environmental salinity (‰)	No. of raw reads	No. of filtered reads	No. of valid reads	Proportion of valid reads (%)	BioSample accession no.
ELJF1	Qinghai Lake	11.17	19,627	17,431	13,174	67.12	SAMN18489513
BHH1	Buha River	0.56	74,711	69,342	30,775	41.19	SAMN18489514
BHH2	Buha River	0.56	70,164	64,572	55,426	78.99	SAMN18489515
BHH3	Buha River	0.56	42,489	39,283	22,929	53.96	SAMN18489516
BHH4	Buha River	0.56	72,942	68,035	56,276	77.15	SAMN18489517
BHH5	Buha River	0.56	72,630	67,021	46,734	64.35	SAMN18489518

### Data availability.

Raw reads in this study were deposited in the NCBI Sequence Read Archive (SRA) database (accession number PRJNA717171).
